# Mitani K, Lee JW, Jang JH, et al. Long-term efficacy and safety of romiplostim in refractory aplastic anemia: follow-up of a phase 2/3 study. *Blood Adv*. 2024;8(6):1415-1419.

**DOI:** 10.1182/bloodadvances.2024014868

**Published:** 2024-12-04

Supplemental Data: in supplemental Table 3, in the row corresponding to Patient #18, under “Duration of platelet response,” “W614” should read “W6-14.”

In supplemental Figure 4, “Number of patients” should read “Number at risk.”

The corrected supplemental Data file is attached to the online version of this erratum.

## Supplementary Material

Supplemental Methods, References, Tables, and Figures

