# Supplementary material for: Mitani K, Lee JW, Jang JH, et al. Long-term efficacy and safety of romiplostim in refractory aplastic anemia: follow-up of a phase 2/3 study. Blood Adv. 2024;8(6):1415-1419
Source: Blood Adv. 2024 Dec 4;8(23):6097. doi: 10.1182/bloodadvances.2024014868 (PMC11652749; doi:10.1182/bloodadvances.2024014868)

Long-term efficacy and safety of romiplostim in refractory aplastic anemia: Follow-up of a phase 2/3 study

Short title: Long-term romiplostim in refractory AA

Kinuko Mitani, Jong Wook Lee et al.

Supplementary methods

Patients

Eligible patients included adults (≥20 and ≥19 years in Japan and Korea, respectively) with AA and thrombocytopenia (platelet count ≤30 × 10^9/L) who were refractory to IST (ATG plus cyclosporin or cyclosporin monotherapy). Patients who were ineligible for ATG treatment due to older age or co-morbidity and refractory to treatment of cyclosporin for at least 6 months were allowed to enroll in this study. Twenty-seven patients who completed the week 53 assessment in the evaluation study and gave written informed consent were enrolled in the extension study.

Study design

The study design is illustrated in supplemental Figure 1. Briefly, this study followed the phase 2/3, multicenter, open-label interventional study of the efficacy and safety of romiplostim, which is referred to here as the "evaluation study". In the evaluation study, romiplostim at a dose of 10 µg/kg was administered subcutaneously once a week for 4 weeks (Weeks 1–4), followed by a titration period with 1-step titration to 5, 10, 15, or 20 µg/kg once weekly up to 52 weeks (Weeks 5–52) depending on platelet response, and a washout period (Weeks 52–56). Romiplostim was started from week 56 in this extension study.

Dose reduction or interruption of romiplostim was performed similar to the evaluation study.[1] During the extension study, the investigators may increase the dose of romiplostim or

restart romiplostim if the patient's platelet count of >30 x 10^9/L, hemoglobin level of >9.0 g/dL, and neutrophil count of >0.5 x 10^9/L could not be maintained after dose reduction or discontinuation of romiplostim. In patients taking 5 μg/kg, romiplostim was discontinued if trilineage hematopoiesis and transfusion independence were maintained for ≥4 weeks; the dose was reintroduced at 5 μg/kg or increased by 1-step either when platelets were <30 × 10^9/L, hemoglobin was <9.0 g/dL, or neutrophils were <0.5 × 10^9/L. For 10-, 15-, or 20-μg/kg doses, romiplostim was reduced by 1-step in patients with trilineage hematopoiesis and transfusion independence for ≥8 weeks; the dose was further reduced by next step in patients whose hematological response was maintained for a further 4 weeks. Romiplostim was suspended when platelet counts exceeded 400 × 10^9/L, or safety issues such as adverse events (AEs) occurred. In cases of the latter, the investigator/sub-investigator may discontinue romiplostim at any time or reduce the dose by 1-step at their own discretion.

Upon the initiation of the extension study at week 56, patients received the same dose of romiplostim as they had received on week 52. Of the 4 patients where romiplostim administration had been suspended before the end of week 52, it was restarted in 3 patients at 5 μg/kg, in line with the evaluation study protocol, while the 4th patient who had responded to romiplostim was observed without romiplostim after entering the extension study. The extension period ended when romiplostim became commercially available for AA in the study country or at the end of June 2020 (end of the extension period), whichever occurred first. Note that 53 weeks is counted as one year in these analyses.

The study was approved by the Institutional Review Board at each study center. The study adhered to the Declaration of Helsinki, national laws and regulations, and the International Conference on Harmonisation of Good Clinical Practice guidelines.

Study measures and outcomes

Measures and outcomes included in this analysis were as follows: romiplostim exposure duration and doses administered up to the end of the extension period; changes in platelet, reticulocyte, and neutrophil count, and hemoglobin level during the extension period; hematological response to romiplostim at 2, 3, and 3.5 years; proportion of transfusion-dependent patients, which were defined as 'those who receive platelet and/or red blood cell transfusions at least once in an 8 week period'; and time to hematological response.

"Any sustained hematologic response after discontinuation of romiplostim" was defined as the response that was maintained without romiplostim for ≥6 months (≥24 weeks) until the end of study. Patients were considered to be "secondary refractory" when 1) any hematologic response (platelets, erythrocytes, neutrophils) was observed for ≥8 weeks but disappeared, and did not return to the previous levels despite continued administration of romiplostim at a dose of 20 μg/kg (maximum dose) (defined as relapsed on romiplostim), or 2) any hematologic response observed for ≥8 weeks but disappeared upon the tapering or cessation of the treatment, and did not return to the previous levels despite re-initiation of romiplostim at a dose of 20 μg/kg (defined as failure to respond to second romiplostim).

Platelet response was defined as an increase of $\geq 20 \times 10^9$/L above the baseline value (at the start of the evaluation study); or an increase of $\geq 10 \times 10^9$/L and by ≥100% from baseline; or no platelet transfusion for 8 weeks in patients who had received platelet transfusions in the 8 weeks prior to the first romiplostim dose. Erythrocyte response was defined as an increase in hemoglobin to ≥9.0 g/dL without red blood cell (RBC) transfusion in patients with pre-treatment hemoglobin of <9.0 g/dL; or cumulative RBC transfusion volume reduction of ≥4 units (1 unit = 200 mL) for 8 weeks in those who had received transfusion during the 8 weeks prior to the first romiplostim dose. Neutrophil response was defined as an increase in neutrophils of ≥100% in patients with baseline neutrophils of $<0.5 \times 10^9$/L; or an increase of $\geq 0.5 \times 10^9$/L over baseline in

patients with baseline neutrophils of 0.5 to 1 × 10^9/L. Trilineage response was considered in patients achieving concurrent platelet, erythrocyte, and neutrophil responses.

Safety

Incidences of AEs and drug-related AEs, laboratory values, vital signs, changes in the 12-lead electrocardiogram, other safety parameters were evaluated. Bone marrow examinations were conducted at Weeks 56, 108, and 160 to identify patients who developed chromosomal abnormalities, and optional bone marrow examinations were conducted at Weeks 82, 134, and 186. Chromosomal abnormalities in chromosome 7, such as -7/7q-, were screened using the G-banding cytogenetic analysis or fluorescence *in situ* hybridization technique. If -7/7q- or other chromosomal abnormalities were detected, patients were withdrawn from the study at the discretion of the medical officer, coordinating investigator, and data monitoring committee. During treatment, patients were monitored to identify any cases of transformation to acute myeloid leukemia (AML) and/or myelodysplastic syndromes (MDS).

Statistical analysis

Categorical data were summarized using frequency and percentage, and continuous variables using descriptive statistics (number of patients, mean, standard deviation, minimum, median, and maximum). Kaplan–Meier analysis was used to calculate the time to hematological response. Statistical tests were 2-sided, with a significance level of 5%. SAS version 9.4 (SAS Institute, Tokyo, Japan) was used to conduct the analyses.

Data sharing statement

All data including study participant data, data dictionary, statistical analysis plan, and informed consent will not be shared. The protocol will be provided on reasonable written request.

Supplementary results

Patient characteristics

The disposition of patients is described in supplementary figure 2. The 27 patients included 25 responders in any hematopoietic lineage and 2 non-responders. The main characteristics of 27 patients at enrollment of the evaluation study and hematologic parameters at the beginning of the extension study are summarized in supplementary Table 1. Most patients were female (19 of 27, 70.4%) and had a median (range) age of 45 (20-78) years. Seven (25.9%) and 3 (11.1%) of 27 patients had severe and very severe AA, respectively. The median (quartile [Q1, Q3]) disease duration was 8.7 (4.0, 14.1) years, and 21 (77.8%) and 4 (14.8%) patients required transfusions at the beginning of the evaluation and extension studies, respectively.
Twenty-three, 7, and 6 patients remained in this study at 2, 3, and 3.5 years, respectively. Seven patients dropped out of the study because of a lack of hematological response (n = 4, one had no response at the start of the extension study), development of chromosomal abnormality (n = 1), investigator's decision (n = 1), and withdrawal of consent (n = 1).

Dose of romiplostim during long-term treatment

While the proportion of patients receiving the maximum dose of 20 μg/kg decreased, that of romiplostim-free patients increased during the study period.

Study outcomes and measures

The mean (standard deviation) values of hematologic parameters at baseline, and Weeks 53 and 168 were 13.9 (5.9) × 10^9/L (n = 31), 44.5 (32.8) × 10^9/L (n = 26), and 51.4 (19.8) × 10^9/L (n = 5) for platelet counts, 7.5 (1.9) g/dL (n = 22), 11.4 (2.2) g/dL (n = 24), and 12.7 (0.6) g/dL (n = 5) for hemoglobin levels, and 0.95 (0.51) × 10^9/L (n = 31), 1.81 (1.08) × 10^9/L (n = 27), and 1.53 (0.50) × 10^9/L (n = 5) for neutrophil counts, respectively. The respective changes of platelet

counts, hemoglobin levels, and neutrophil counts from baseline were 34.8 (36.6) × 10^9/L (n = 25), 4.2 (2.7) g/dL (n = 16), and 0.80 (0.66) × 10^9/L (n = 27).

The respective numbers of platelet and erythroid responders to romiplostim were 20 of 27 (74.1%) and 21 of 27 (77.8%) at 1 year, 16 of 22 (72.7%) and 17 of 22 (77.3%) at 2 years, and 6 of 6 (100%) and 5 of 6 (83.3%) at 3 years.

Safety

In G-banding analysis of bone marrow cells, 2 patients had chromosomal abnormalities during the extension study (**Table S5**), including t(11;17)(q23;q12) or t(11;17)(q23;q21) after transient occurrence of hyperdiploid karyotype and add(10)(p11.2). The patient that developed t(11;17)(q23;q12) or t(11;17)(q23;q21) (five metaphases among 20) at Week 108 continued to receive 15 µg/kg of romiplostim at Week 108, and the dose was increased to 20 µg/kg at Week 117 because of poor responsiveness. The clone carrying t(11;17)(q23.2;q21.2) or t(11;17)(q23;q21) was expanded (16 of 20 metaphases) at Week 134.

Supplementary discussion

The present study demonstrated the safety and effectiveness of long-term administration of romiplostim in patients with refractory AA, and is a first-of-its-kind study that demonstrated that approximately 15% patients treated with long-term romiplostim maintained their responses for at least 29 weeks after withdrawal of the therapy. During the long-term follow-up period, hematological parameters continued to increase or remained stable in most patients, despite a continuous dose reduction of romiplostim. Previously, a phase II study on romiplostim with a 2-year extension period demonstrated the efficacy of prolonged administration of romiplostim in 30% of patients with refractory AA.[2] However, as that study focused on the optimization of the starting dose of romiplostim, it did not analyze the incidence of sustained response after discontinuation of romiplostim.

The main findings of this follow-up analysis were the long-term effects of romiplostim. The hematological parameters, in patients who achieved response to romiplostim, were either maintained or increased by the administration of the same or reduced dose of romiplostim during the extension period. At 2 years (1 year of the extension study; n = 22), 3 of the 7 patients with no platelet response and 1 of the 6 patients with no erythrocyte response, and 1 of 2 patients with no response in any of 3 lineages of cells at Week 53 finally responded. All patients who remained in the study exhibited responses in the trilineage of cells (n = 5) or in the platelet (n = 1). The cumulative incidence of the trilineage response to romiplostim reached a plateau at 55% within 1 year of treatment and remained stable over nearly 2.5 years thereafter.

Four patients (15%) maintained any sustained hematologic response even after discontinuation of romiplostim. There was a tendency toward higher reticulocyte counts at baseline in patients with any sustained hematologic response after romiplostim discontinuation compared with those who required romiplostim to maintain any sustained hematologic response. However, probably because of the small number of patients, this difference was not statistically significant (*P* = .087) and further studies with larger sample sizes are needed to confirm these findings.

At the beginning of the evaluation study, 74% of the patients were dependent on transfusions, which decreased to none of the patients requiring transfusions by the end of the study. Although the percentages of platelet (74.1%) and erythroid (77.8%) responders to romiplostim at the beginning of the extension study remained similar (72.7% and 77.3%) at 2 years, they rose to 100% and 83.3% at 3.5 years. The decrease in the proportion of transfusion-dependent patients and the increase in the proportion of responders in different lineages indicate the persistence of the efficacy of romiplostim over the long term.

The reasons for delayed effects of romiplostim on hematopoiesis that appeared in some patients remain unknown. Their direct action only on HSCs cannot explain their late effect on hematopoiesis. Nakamura-Ishizu et al.[3] demonstrated that a membrane protein C-type lectin-like

receptor 2 that is expressed on megakaryocytes and associates with podoplanin on the bone marrow fibroblastic reticular cells mediates secretion of thrombopoietin from megakaryocytes and constitutes the megakaryocyte niche, where quiescence and the repopulation potential of HSCs are maintained. Reconstitution of the megakaryocyte niche to promote self-sustaining hematopoiesis could be one of the mechanisms underlying stimulation of hematopoiesis by thrombopoietin-receptor agonists. In patients who showed late responses to romiplostim, a longer time might have been required to re-establish the megakaryocyte niche in the bone marrow to stimulate HSCs.

During the extension study, the dose of romiplostim was on a downward trend in most patients, in parallel with the appearance of its effects. The median dose of romiplostim at the end of the prior study (at Week 52) was 20 μg/kg, and was 15 μg/kg at 2 years and 7.5 μg/kg at 3.5 years. However, 5 patients lost response to romiplostim despite having received the maximum dose (20 μg/kg). The reason for the loss of response is unclear; HSCs of these patients may have undergone secondary genetic changes that lower sensitivity to romiplostim. Alternatively, an immune attack against hematopoietic stem cells may have flared up for some reason during therapy, which could not be suppressed by romiplostim. There is a need to address the issue of secondary unresponsiveness using a larger sample size.

It is worth noting that in this study most patients had non-severe AA (63.0%) at the time of initial diagnosis. Previous studies have indicated that the rates of non-severe AA are 54.2% and 50.2% in Japan and Korea, respectively.[4,5] In contrast, the rate of non-severe AA in the United States has been shown to be 26.4%.[6] Although our study cohort included non-severe AA patients that accounted for 63.0% of the participants, the high response rate (77.8%) to long-term treatment with romiplostim at any given time suggests that romiplostim may have potent hemato-stimulatory effect in the treatment of AA.

Most AEs related or possibly related to romiplostim were grade 1 or 2. Notably, in the follow-up bone marrow examinations, none of the patients experienced transformation to

AML/MDS. However, newly developed chromosomal abnormalities were identified in two patients during the extension study. Although the clinical significance of the abnormalities (46,XX,t(11;17) and 46,XX,add(10) detected in the 2 patients) is obscure, monitoring is required when administering romiplostim to patients with AA for long periods of time. A low incidence of secondary AML/MDS has also been reported in Japanese patients with refractory AA treated with eltrombopag.[7] Previous epidemiology studies revealed the incidence of secondary AML/MDS was as low as 3% in Japanese and Korean AA patients.[5,8,9] Thus, the lower incidence than those reported in patients treated in the United States and Europe with eltrombopag may be attributed to ethnic factors.[10]

This follow-up analysis has several limitations. Responses to romiplostim in patients who left the study early due to the market approval of romiplostim for AA could not be analyzed in this study. Although this extension study was interventional and intended to observe the long-term effects of romiplostim treatment, influences of other factors, such as the effects of concomitant medications and complications, were not considered. The small number of patients made it difficult to make statistical analysis to predict the efficacy of romiplostim. As this study was a collaborative study between Japan and Korea, the generalizability of these findings is difficult. Additionally, cyclosporin and danazol were allowed to use throughout the study.

Further observation is necessary to determine the influence of romiplostim on the long-term prognosis of AA patients.

References

1. Jang JH, Tomiyama Y, Miyazaki K, et al. Efficacy and safety of romiplostim in refractory aplastic anaemia: a phase II/III, multicentre, open-label study. *Br J Haematol.* 2021;192(1):190-199.

2. Lee JW, Lee SE, Jung CW, et al. Romiplostim in patients with refractory aplastic anaemia previously treated with immunosuppressive therapy: a dose-finding and long-term treatment phase 2 trial. *Lancet Haematol.* 2019;6:e562-572.
3. Nakamura-Ishizu A, Takubo K, Kobayashi H, Suzuki-Inoue K, Suda T. CLEC-2 in megakaryocytes is critical for maintenance of hematopoietic stem cells in the bone marrow. *J Exp Med*. 2015;212(12):2133-2146.
4. Ohta A, Shimada N. Descriptive epidemiology of severe aplastic anemia: Analysis of clinical survey personal records [Japanese]. https://mhlw-grants.niph.go.jp/system/files/2017/172051/201711077A_upload/201711077A0013.pdf (Accessed September 15, 2023)
5. Kim SY, Lee JW, Lee SE, et al. The characteristics and clinical outcome of adult patients with aplastic anemia and abnormal cytogenetics at diagnosis. *Genes Chromosomes Cancer.* 2010;49(9):844-850.
6. Patel BJ, Barot SV, Kuzmanovic T, et al. Distinctive and common features of moderate aplastic anaemia. *Br J Haematol.* 2020;189(5):967-975.
7. Yamazaki H, Ohta K, Iida H, et al. Hematologic recovery induced by eltrombopag in Japanese patients with aplastic anemia refractory or intolerant to immunosuppressive therapy. *Int J Hematol*. 2019;110(2):187-196.
8. Kojima S, Hibi S, Kosaka Y, et al. Immunosuppressive therapy using antithymocyte globulin, cyclosporine, and danazol with or without human granulocyte colony-stimulating factor in children with acquired aplastic anemia. *Blood.* 2000;96(6):2049-2054.
9. Teramura M, Kimura A, Iwase S, et al. Treatment of severe aplastic anemia with antithymocyte globulin and cyclosporin A with or without G-CSF in adults: a multicenter randomized study in Japan. *Blood.* 2007;110(6):1756-1761.

10. Desmond R, Townsley DM, Dumitriu B, et al. Eltrombopag restores trilineage hematopoiesis in refractory severe aplastic anemia that can be sustained on discontinuation of drug. *Blood*. 2014;123(12):1818-1825.

Supplementary tables

Table S1. Patient characteristics at baseline and hematologic parameters at the beginning of extension study

**Characteristic, n (%)*** **Category**	**Total (N = 27)** **at baseline**	**Total (N = 27)** **at Week 53**
**Sex**		
Female	19 (70.4)	
**Age (years)**		
Median (Q1, Q3)	45 (35.0, 56.0)	
**Severity of aplastic anemia**		
Non-severe	17 (63.0)	
Severe	7 (25.9)	
Very severe	3 (11.1)	
**Disease duration (years)**		
Median (Q1, Q3)	8.7 (4.0, 14.1)	
**Platelet count (× 10^9/L)**		
Median (Q1, Q3)	14.0 (8.0, 18.0)	36.0 (21.0, 54.0)
**Hemoglobin concentration (g/dL)**	n=20	n=27
Median (Q1, Q3)	6.8 (6.2, 7.7)	10.9 (8.1, 13.1)
**Neutrophil count (×10^9/L)**		

Median (Q1, Q3)	0.83 (0.53, 1.09)	1.64 (1.03, 2.46)
**Reticulocyte count (× 10^9/L)**		
Median (Q1, Q3)	50.2 (27.3, 60.0)	78.5 (59.4, 97.0)
**RBC and platelet transfusions**		
No blood transfusion	6 (22.2)	23 (85.2)
RBC transfusion	8 (29.6)	2 (7.4)
Platelet transfusion	3 (11.1)	0 (0.0)
RBC and platelet transfusions	10 (37.0)	2 (7.4)
**Prior treatment**		
ATG + cyclosporin	18 (66.7)	
Cyclosporin monotherapy	8 (29.6)	
G-CSF	2 (7.4)	
Other	22 (81.5)	

G-CSF indicates granulocyte colony-stimulating factor; Q, quartile; RBC, red blood cell; and SD, standard deviation.

*Unless otherwise specified.

Table S2. Background factors of patients who achieved any sustained hematologic response after discontinuation of romiplostim.

Patient characteristics					Baseline				Blood transfusion		At Week 53				Time of response and discontinuation	Duration of discontinuation (weeks)
Patient No. in Figure S5	Age (years)	Sex	Disease duration (years)	Severity of AA*	Platelet count (× 10^9/L)	Hb concentration (g/dL)	Neutrophil count (× 10^9/L)	Reticulocyte count (× 10^9/L)	Platelets	RBCs	Platelet count (× 10^9/L)	Hb concentration (g/dL)	Neutrophil count (× 10^9/L)	Reticulocyte count (× 10^9/L)		
#4	20	F	12.0	NSAA	12	6.4	0.83	82.3	N	Y	54	12.1	2.58	100.0	W78-188 (EoS)	111
#10	35	F	10.2	NSAA	23	8.4	1.76	57.5	N	N	41	11.6	1.19	78.5	W121-149 (EoS)	29
#15	50	F	2.3	SAA	28	10.5	1.62	93.8	N	N	63	12.8	3.29	100.1	W60-138 (EoS)	79
#26	43	M	0.5	NSAA	11	N/A	1.28	50.2	Y	Y	92	12.5	5.11	83.5	W51-92 (EoS)	42

EoS indicates end of study; Hb, hemoglobin; NSAA, non-severe aplastic anemia; SAA, severe aplastic anemia; and W, Week.

*At the diagnosis of AA.

Table S3. Patients with AA who relapsed on romiplostim and/or failed to respond to secondary romiplostim treatment

Patient characteristics					Baseline				Concomitant drug			Duration of platelet response	Minimum platelet count / maximum platelet count during platelet response (× 10^9/L)	Duration of RBC response	Minimum Hb concentration / maximum Hb concentration during RBC response (g/dL)	Maximum dose of romiplostim	Reason for discontinuation
Patient No. in Figure S5	Age (years)	Sex	Disease duration (years)	Severity of AA*	Platelet count (× 10^9/L)	Hb concentration (g/dL)	Neutrophil count (× 10^9/L)	Reticulocyte count (× 10^9/L)	CsA	Metenolone	Danazol						
#18[†]	46	F	30.0	SAA	18	6.4	1.09	33.9	Y	N	Y	W6-14, W16-41, W44, W47, W63-67, W76	36/50	W7-122	7.0/12.5	20 μg/kg[§]	No efficacy
#21[†]	78	F	7.6	VSAA	8	9.1	0.95	114.7	Y	Y	N	W6-104	16/70	None	N/A	20 μg/kg	Marketing approval
#23[†]	51	F	24.7	NSAA	14	N/A	0.78	29.5	Y	Y	N	W26-29, W38-83, W92-100	5/18	W10-46, W57, W60	6.2/7.9	20 μg/kg	Investigator's decision
#25[†]	65	F	4.4	NSAA	14	7.7	2.05	60.0	Y	N	N	W5–27, W29-53, W64-90	11/50	W9-90	5.0/9.7	20 μg/kg[§]	No efficacy
#27[‡]	75	M	2.4	NSAA	8	4.6	0.90	27.23	Y	Y	N	W17, W19-61	6/114	W1-63	5.5/12.1	20 μg/kg[§]	No efficacy

CsA indicates cyclosporine A; F, female; Hb, hemoglobin; N, no; M, male; N/A, not applicable; VSAA, very severe aplastic anemia; and Y, yes.

*At the diagnosis of AA.

†These patients relapsed on romiplostim; that is, they initially responded to romiplostim but lost response under continuous administration at the maximum dose of 20 μg/kg for more than 8 weeks.

‡This patient failed to respond to secondary romiplostim treatment; that is, he discontinued romiplostim after achieving response, then did not respond to re-initiation with the maximum dose.

§After the response disappeared, the dose was increased to 20 μg/kg. However, no response was obtained after 8 weeks of administration; therefore, these patients discontinued romiplostim according to the discontinuation criteria.

Table S4. Summary of the AEs in the extension study

Severity of AE	Possibly related			Related		
	No. of events	No. of patients	Incidence* (%)	No. of events	No. of patients	Incidence* (%)
Grade 1	6	4	14.8	1	1	3.7
Grade 2	3	2	7.4	1	1	3.7
Grade 3	1†	1	3.7	0	0	0.0
Grade 4	0	0	0.0	0	0	0.0
Grade 5	0	0	0.0	0	0	0.0

AE indicates adverse event.

*Incidence proportion (%): Number of patients with AEs/number of patients in safety analysis (N = 27) × 100.

†One possibly related AE was weight increase.

Table S5. Details of chromosomal abnormalities observed in the extension study population

Patient No. in Figure S5	Time	G-banding	FISH (-7/7q-)
#8	Screening	46,XX[20]	0.0%
	Week 27	46,XX[20]	0.0%
	End of evaluation period (Week 53)	46,XX[20]	0.0%
	Week 108	46,XX	0.0%
	Week 134	46, XX, add(10)(p11.2) [1]/ 46,XX[19]	0.0%
#16	Screening	46,XX[20]	0.0%
	Week 27	53,XX,+3,+4,+14,+16,+17,+19,+21[1]/46,XX[8]	0.0%
	End of evaluation period (Week 53)	46,XX [12]	0.0%
	Week 108	46,XX,t(11;17)(q23;q12)or t(11;17)(q23;q21)[5]/46,XX[15]	1.0%
	Week 134	46,XX, t(11;17)(q23.2;q12.2) or t(11;17)(q23.3;q21)[16]/ 46,XX[4]	0.0%

FISH indicates fluorescence *in situ* hybridization .

Supplementary figures

Figure S1. Study design

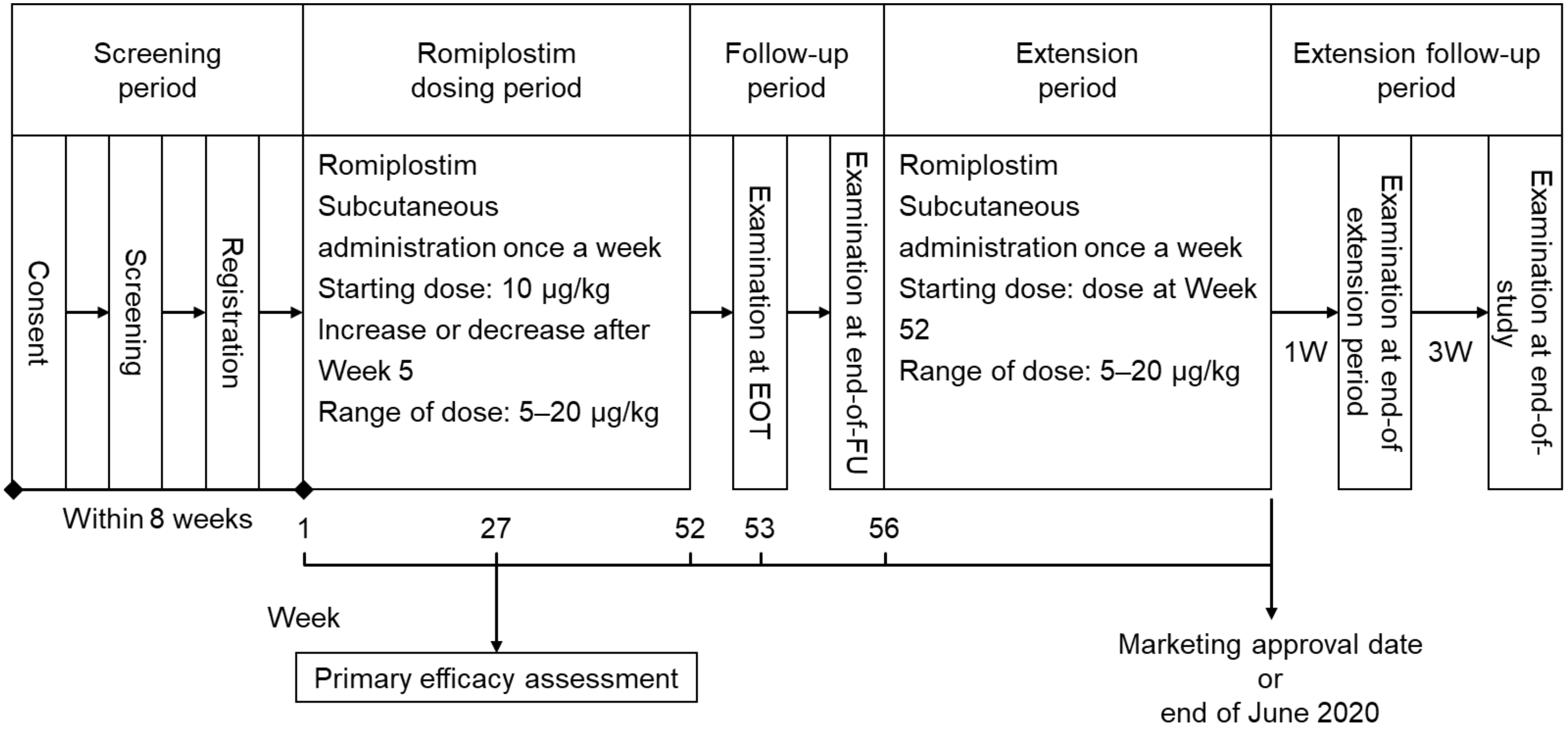

Figure S2. Patient disposition

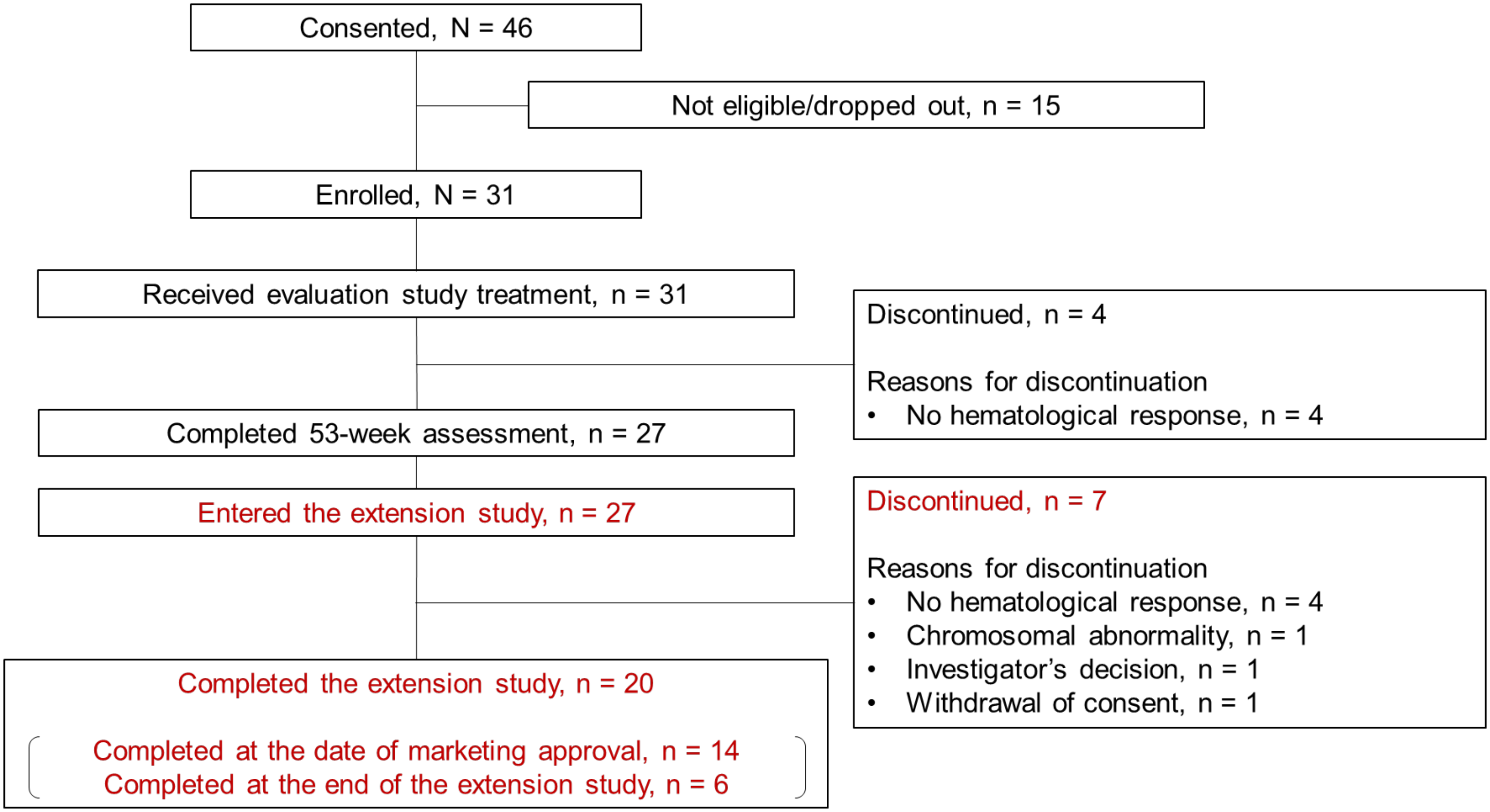

*Red indicates extension study.

Figure S3. Romiplostim doses administered to patients each week during the entire period

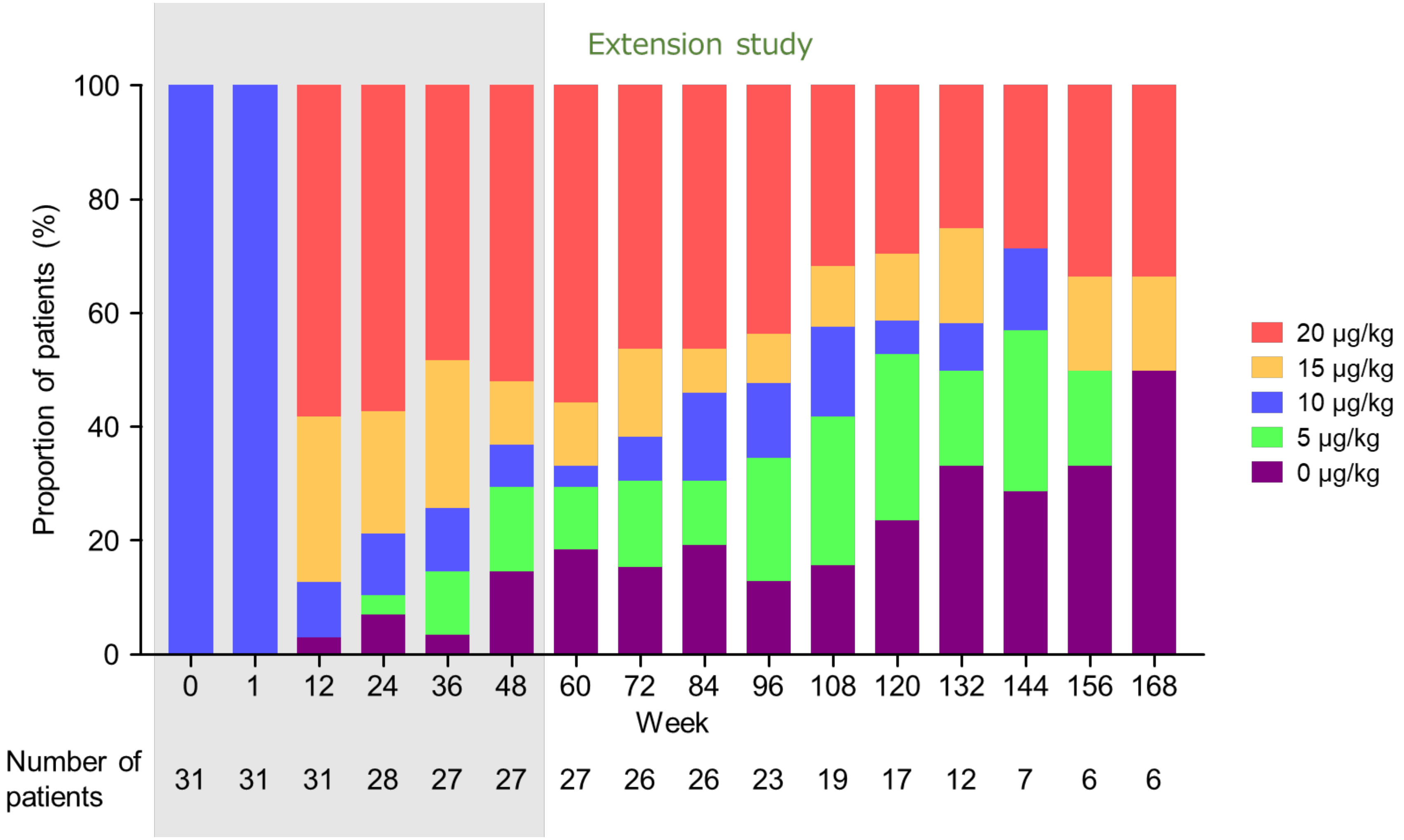

Figure S4. Kaplan–Meier curve of trilineage response during the entire period

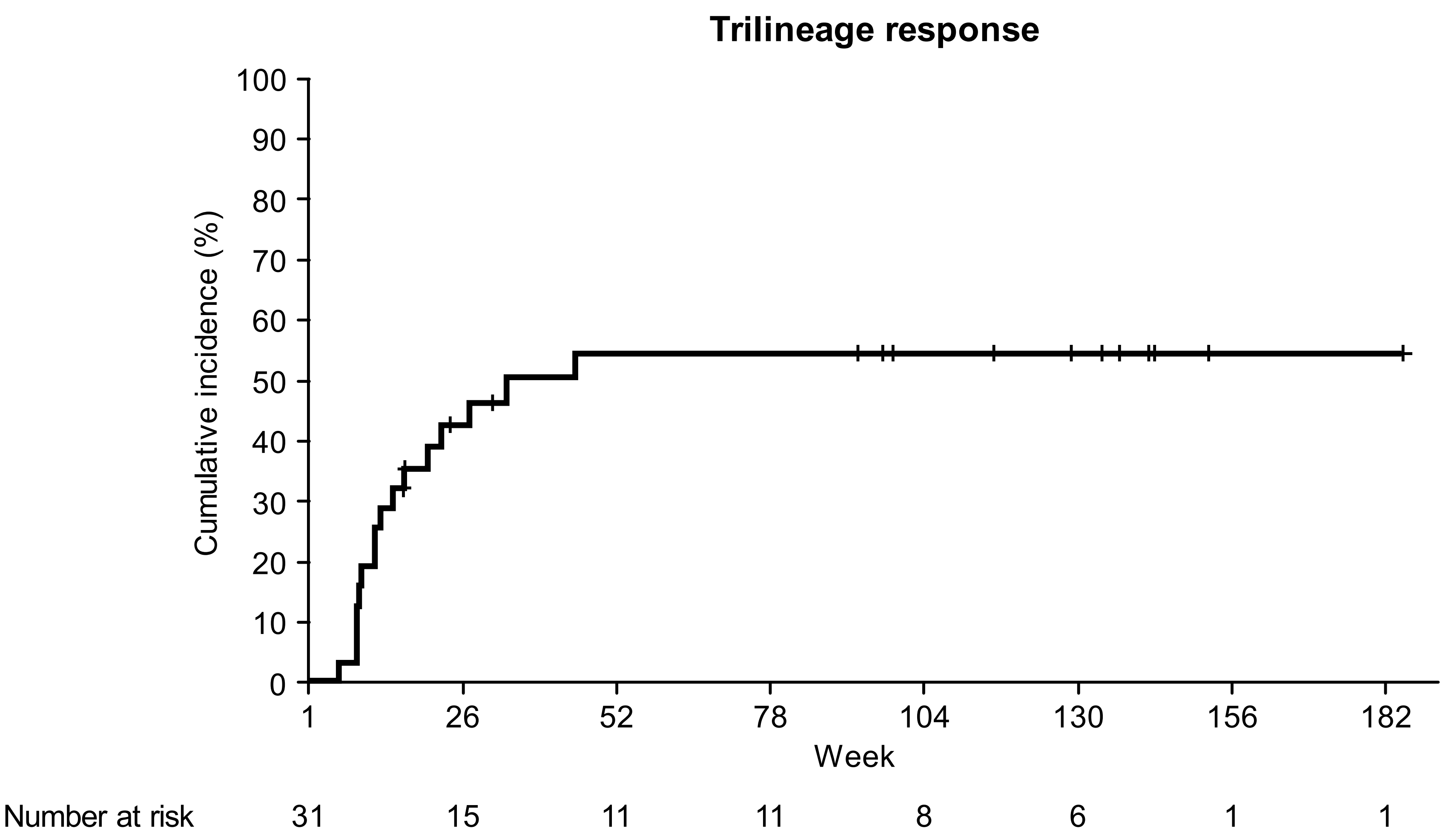

Figure S5. Swimmer plot of patients with trilineage response at the onset of hematological response

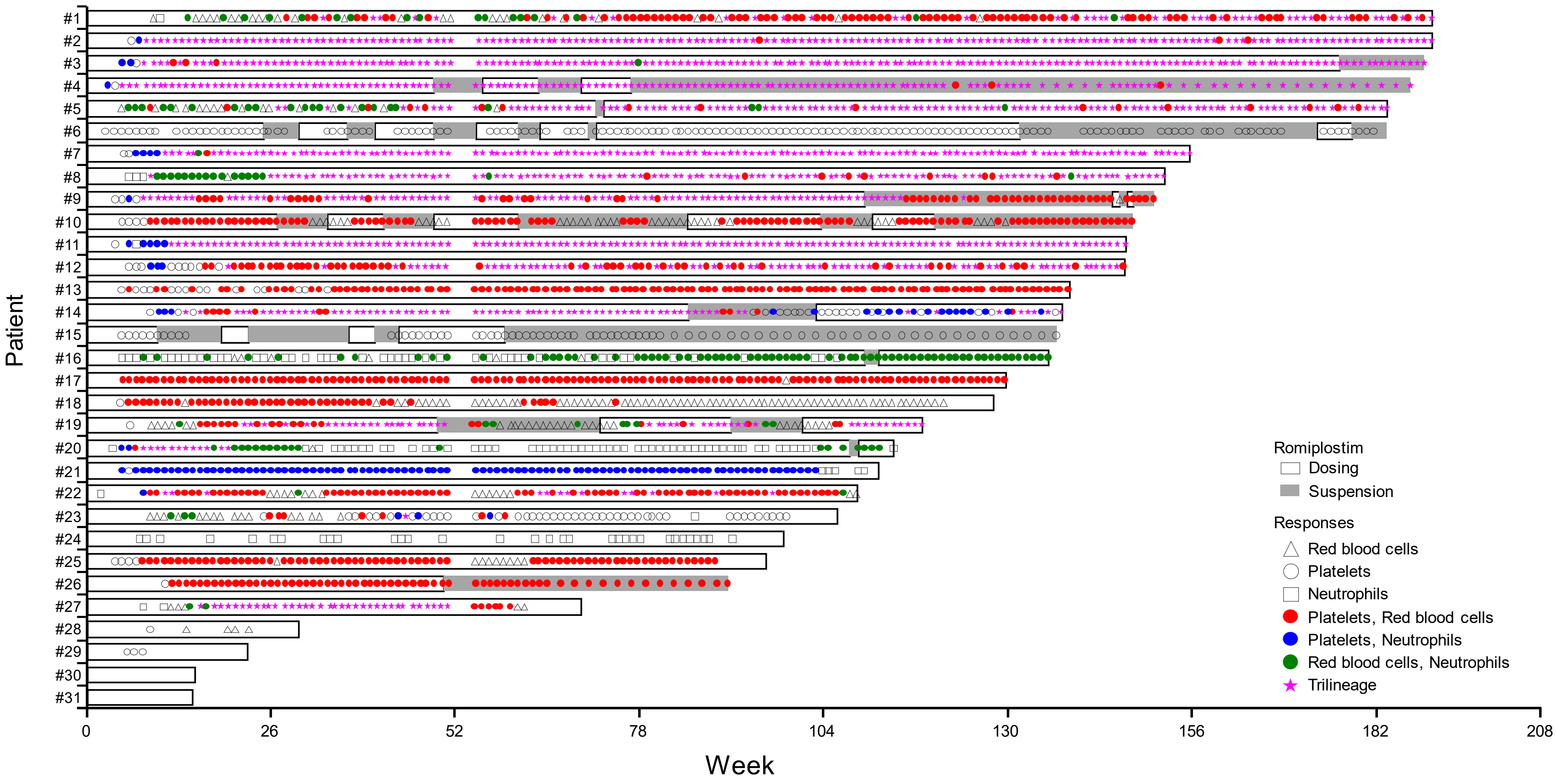

Figure S6. Proportion of transfusion-dependent patients during the entire period (182 weeks)

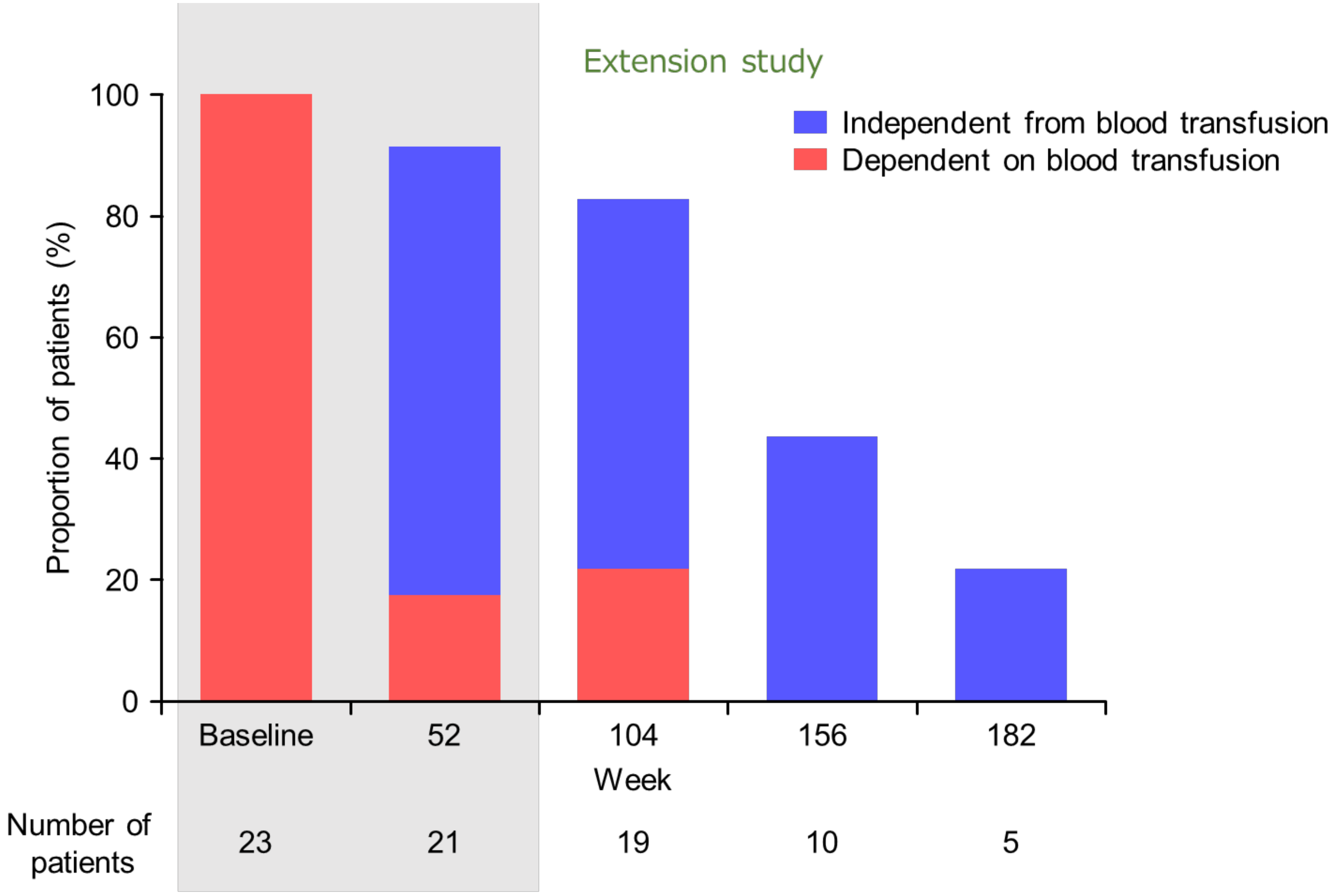

Supplement: Supplemental Methods, References, Tables, and Figures [file BLOODA_ADV-2024-014868-mmc1.pdf]
